# Opium addiction and severity of coronary artery disease: a case-control study

**Published:** 2010

**Authors:** Mohammad Masoumi, Armita Shahesmaeili, Ali Mirzazadeh, Marjan Tavakoli, Arghavan Zia Ali

**Affiliations:** a Associate Professor of Cardiology, Department of Cardiology and Physiology Research Center, Kerman University of Medical Sciences, Kerman, Iran.; b Clinical Research Department, Physiology Research Center, Kerman University of Medical Sciences, Kerman, Iran; c Physiology Research Center, Kerman University of Medical Sciences, Kerman, Iran; d Student of Medicine, Kerman University of Medical Sciences, Kerman, Iran

**Keywords:** Coronary Artery Disease, Atherosclerosis, Opium, Risk Factor

## Abstract

**BACKGROUND:**

Coronary artery disease (CAD) is a leading cause of morbidity and mortality in the world. Determination of the risk factors and high risk groups plays an important role in the prevention and controlling programs. The present study aims to determine the relationship between opium consumption and severity of CAD.

**METHODS:**

In this hospital based case-control study, 299 patients who were candidates for coronary angiography from 2006 to 2007 were recruited. The patients’ history of opium addiction was taken. Based on their history, they were categorized into three groups: non users, occasional users and current users. The relationship between opium addiction and severity of CAD was analyzed by a multiple logistic regression model, STATA v.10.

**RESULTS:**

According to angiographic data, patients were divided into 3 groups: 84 patients (28.09%) as non coronary artery disease, 81 patients (27.09%) as mild CAD and the remaining 134 patients (44.82%) as severe CAD. Univariate analysis showed that current opium users had higher odds of severe CAD compared with non users. Multivariate analysis showed a significant relationship between age, diabetes, hyperlipidemia, gender and severity of coronary artery stenosis.

**CONCLUSIONS:**

The findings indicated that current opium users - in comparison with non users - have a higher risk for severe CAD. But it is roughly confounded by other co-factors such as cigarette smoking, age and sex. A dose-response was noticed between the type of opium consumption and the severity of CAD.

Coronary artery disease (CAD) is the leading cause of morbidity and mortality in the developed countries. It is also increasing in developing nations to an alarming rate.^1^ CAD is the leading cause of death and disability among Iranian population too.^2^ It seems that about 50% of annual deaths in Iran are due to CAD.^3^

Clinical manifestations of CAD are most often due to atherosclerosis of epicardial coronary artery. The main risk factors of atherosclerosis are: dyslipidemia, diabetes mellitus, hypertension, smoking, immobility and obesity. Other risk factors are aging, male gender, menopause and positive family history of CAD.^4^,^5^ The role of other potential risk factors in the development of CAD needs to be investigated.

Opium abuse is the major public health problem in all societies including Iran, where it is considered the most common drug abuse. In some societies especially in Asia and Middle East, there is a belief that opium consumption has a preventive effect against cardiovascular diseases, hypertension and diabetes.^6^ This belief is possibly the main reason for high rate of opium addiction in these societies. Some studies indicate that opium consumption can modulate some risk factors of CAD, but others believe that it is harmful.^6^–^8^ Marmer et al in 2004 showed that long exposure to opiates among autopsy samples was associated with decreased severity of CAD and its fatal consequence.^8^ But another study conducted by Mohammadi et al showed that in animal model, opium consumption exacerbated the atherosclerosis plaque formation by hypercholesterolemia.^6^ Also, Sadeghian et al in 2007 showed that opium consumption was an important risk factor of CAD and amount of consumption was associated with severity of the disease.^7^ Another study in Iran showed that the prevalence of opium addiction among myocardial infarction patients was higher than normal population.^9^

In the review of literature, little focus has been placed on the relation between opium addiction and severity of CAD. The objective of this study was to determine the relationship between the opium consumption and the severity of CAD. The interaction effect and the confounding of the other classic risk factors were considered.

## Methods

In this hospital based case-control study, 299 patients who were candidates for coronary angiography from 2006 to 2007 were consequently recruited. The candidates for coronary angiography were included in this study based on the medical history, physical examination and paraclinic data (echocardiography, exercise test and thallium scan).

Before further evaluations, the patients with the valvular heart disease and congenital heart disease were excluded. All the remaining subjects were asked to sign an informed consent which explained all the aspects of study as well as permission for coronary angiography implementation. The protocol has been registered and approved by the local committee of Kerman Physiology Research Center. Before the angiography, important demographic data and potential confounders including sex, age, and history of cigarette smoking were collected by a trained examiner through a short interview. Those who reported smoking regularly during past 3 years were grouped as current smokers; those who had smoked regularly for at least 3 years but not during the last year were grouped as former smokers; and those who had never smoked were grouped as non smokers.^10^ Co-morbidities such as diabetes (FBS ≥ 126 mg/dl or/and under hypoglycemic treatment), hypertension (blood pressure > 140/90 mmHg or/and under hypertensive treatment), obesity (overweight: BMI 25-30 kgm^-2^, obese: BMI ≥ 30 kgm^-2^), hyperlipideima (cholesterol/triglyceride ≥ 200 mg/dl or/and under treatment) and the positive family history of coronary heart diseases, were assessed.

All the patients were interviewed based on the DSM-IV criteria for opium dependency.^11^ According to the duration of opium consumption, they were categorized into three groups, “non users” who had no opium consumption, “occasional users” who were not dependent but irregularly used opium and “current users” who were dependent and have regularly consumed opium for at least recent three years.

The coronary angiography was performed for all patients by cardiologists, using the Judkins technique. All the angiographic films were checked by a well-experienced cardiologist.

Patients were divided into three groups with respect to their anatomical extent of disease: those without any atherosclerotic plaque in “non CAD” group, patients with non significant stenosis of major coronary arteries (less than 50% luminal diameter stenosis) and those with one-vessel disease (more than 50% stenosis) in “mild CAD” group and the remaining patients with two or three vessel disease in “severe CAD” group.

### 

#### Statistical Analysis

In order to explore the relationship between opium consumption (as the predictor) and the severity of coronary atherosclerosis, univariate and multiple ordinal logistic regression models were applied. Other demographic variables and potential confounders (sex, age, cigarette smoking, co-morbidities such as diabetes, hypertension, obesity, hyperlipideima and the positive family history of coronary heart diseases) were entered in the model as predictor variables and the control of these variables were confounders. The crude and adjusted OR was reported. P values less than 0.05 was considered as significant level.

## Results

The characteristics of the study subjects in the three groups were compared by bivariate analysis and presented in table 1. The only sig-nificant factor among different CAD groups was sex (p < 0.01). There were no significant differences between the CAD groups regarding age (p = 0.31), cigarette smoking (p = 0.16), past medical history (p: 0.13 to 0.51) and obesity (p = 0.90) (Table 1).

**Table 1:** The characteristics of the subjects by severity of coronary artery atherosclerosis

Variable	Non CAD* (n = 84)	Mild CAD** (n = 81)	Severe CAD*** (n = 134)	P value
Age	51.85 ± 8.52	52.20 ± 10.22	53.74 ± 10.04	0.31
Male	32 (38.10%)	48 (59.26%)	89 (66.42%)	0.000
Cigarette smoking				0.16
None	60 (71.43%)	56 (69.14%)	79 (58.96%)	
Former	4 (4.76%)	3 (3.70%)	14 (10.45%)	
Current	20 (23.81%)	22 (27.16%)	41 (30.60%)	
Medical history				
Diabetes	14 (16.67%)	18 (22.22%)	38 (28.36%)	0.13
Hypertension	29 (34.52%)	35 (43.21%)	53 (39.55%)	0.51
CAD in family	18 (21.43%)	22 (27.16%)	41 (30.60%)	0.33
Hyperlipidemia	29 (34.52%)	34 (41.98%)	62 (46.27%)	0.23
Obesity				
None	43 (51.19%)	45 (55.56%)	74 (55.22%)	0.90
Overweight	31 (36.9%)	29 (35.80%)	49 (36.57%)	
Obese	10 (11.90%)	7 (8.64%)	11 (8.21%)	
Opium				
Non users	60(71.4%)	50 (61.7%)	71 (53%)	0.08
Occasional users	12(14.3%)	12 (14.8%)	24 (18%)	
Current users	12(14.3%)	19 (23.5%)	39 (29%)	

* Without any atherosclerotic plaque

** Non significant stenosis of major coronary arteries (less than 50% luminal diameter stenosis) and those with one-vessel disease (more than 50% stenosis)

* With two or three vessel stenosis affected more than 50% of the luminal diameter

CAD: Coronary Artery Disease; BMI: Body Mass Index

Overweight: BMI ≥ 25 kg/m^2^ ; Obese: BMI ≥ 30 kg/m^2^

Generally, 118 (39.5%) cases had history of opium consumption. Among them, more than 90% (106 cases) used opium by inhalation, while the others consumed it orally. About 23% (70 patients) were current opium consumers and 48 (16.05%) cases used it occasionally.

The last row in table 1 shows the different types of consumption regarding the severity of CAD. In a general view, the rate of current users increased constantly by the severity of CAD from 14.3% in the non CAD group to more than 29% in the severe CAD group. The linear trend was statistically significant (p = 0.004). However, the percentage of occasional users did not vary regarding changes in the severity of CAD.

In comparison with those who did not consume opium, occasional users (OR = 1.53, CI 95% 0.84-2.79) and current users (OR = 2.06, CI 95% 1.22-3.49) had higher odds of severe CAD. When the relationship was adjusted for the other potential risk factors (sex, cigarette smoking, diabetes, hypertension, family history of CAD, obesity and hyperlipidemia), occasional users had higher odds of severe CAD compared with the control group (OR = 1.14, CI 95% 0.58-2.22, p = 0.7), while current users had an adjusted (OR = 1.82, CI 95% 0.93-3.58, p = 0.08) (Table 2). Remaining significant factors in the final model were age (OR = 1.02, p = 0.04), male gender (OR = 2.94, p <; 0.001), diabetes (OR = 1.88, p = 0.02) and hyperlpidemia (OR = 1.69, p = 0.03) (Table 2).

**Table 2:** Multivariate Ordinal Logistic Regression analysis of the different factors predicting the severity of CAD, (Crude and Adjusted Odds ratio)

Variable	Crude analysis			Adjusted analysis		
	OR	CI 95%	P value	OR	CI 95%	P value
Opium						
Non users	1	----		1		
Occasional users	1.53	0.84-2.79	0.16	1.14	0.58-2.22	0.70
Current users	2.06	1.22-3.49	0.007	1.82	0.93-3.58	0.08
Age	1.01	0.99-1.03	0.14	1.02	1.00-1.04	0.048
Male	2.38	1.54-3.67	0.000	2.94	1.67-5.18	0.000
Cigarette smoking						
None	1			1		
Former	2.66	1.04-6.81	0.04	1.54	0.56-4.24	0.39
Current	1.41	0.87-2.29	0.15	0.86	0.45-1.63	0.65
Medical history						
Diabetes	1.67	1.009-2.79	0.04	1.88	1.07-3.27	0.02
Hypertension	1.13	0.73-1.73	0.57	1.33	0.81-2.16	0.25
CAD in 1^st^ degree family	1.42	0.93-2.21	0.09	1.60	0.95-2.69	0.07
Hyperlipidemia	1.44	0.93-2.21	0.14	1.69	1.05-2.73	0.03
Obesity						
None	1			1		
Overweight (BMI ≥ 25 kg/m^2^)	0.94	0.60-1.48	0.81	0.83	0.51-1.37	0.48
Obese (BMI ≥ 30 kg/m^2^)	0.71	0.33-1.50	0.37	0.69	0.31-1.52	0.36

## Discussion

The findings indicated that current opium users - in comparison with non users - had a higher risk for severe CAD with the OR = 2.06. Such effect was roughly confounded by other co-factors such as cigarette smoking, age and sex, which were reduced to OR = 1.82 and were not statistically significant. A doseresponse relationship between the type of opium consumption and the severity of CAD was observed, but it was not significant.

More than two third of the non users were free of obvious coronary plaque in angiography, but this rate decreased about ten percentage with higher level of opium consumption. Mild CAD frequency is mostly constant among different opium users, while severe CAD is constantly raised with increase in level of opium consumption. Both non CAD and severe CAD frequency indicated the dose response between the level of opium consumption and the severity of CAD.

In the present study, there was a significant relationship between age, diabetes, hyperlipidemia, male gender and severity of coronary artery stenosis, which is confirmed by other studies. Zeina et al in 2008 reported that the prevalence and severity of CAD among patients with asymptomatic diabetes is higher than non diabetic ones.^12^ Yavuz et al in 2008 also found that diabetes is an important risk factor for severity of CAD.^13^

Drug abuse is one of the most important health problems in the world. In Iran the most frequent substance abuse is opium. In Kerman (a province of Iran), 5.3% of general population and 22.5% of rural population are addicted to opium.^14^ The prevalence of current opium consumption in Fars (another province of Iran) was reported 8.8%.^15^ The high frequency of opium abuse among people is related to a misconception. Some people believe that opium consumption can ameliorate or prevent cardiovascular diseases. But this belief is in contrast with recent clues.^9^

Some studies indicate that opium can interact with some risk factors of atherogenesis. Recent studies have showed that the level of plasma fibrinogen in opium addicted subjects is higher when compared with a control group and hyperfibrinogenemia can prone these patients to the atherogenesis.^1^,^12^,^16^ Opium consumption can also change the lipid profiles. Mohammadi et al showed that in animal model, opium consumption exacerbating the atherosclerosis plaque formation by hypercholesterolemia.^6^ Asgary et al also demonstrated the deleterious effects of opium addiction on cardiovascular risk factors.^17^

Other researches demonstrate an increased activity of coagulative system and decreased activity of fibrinolytic system in opium addicted subjects.^17^,^18^ Naderi et al showed that the level of factor VII in opium addicted patients is higher than non addicted ones.^18^ Another study revealed that the biological activity of Antithrombin III decreases in opium addicts.^19^ These changes in coagulative and fibrinolytic systems can make this population susceptible to atherogenesis.

Opium can also elevate the serum level of some inflammatory biomarkers. Asgary et al reported that the level of Creactive protein (CRP) is significantly higher in opium addiction.^17^ The role of inflammation in the pathogenesis of atherosclerosis is well recognized.^16^,^20^

Beyond its direct effects, opium consumption can be accompanied by specific risky lifestyles such as cigarette smoking, low physical activity and poor nutrition. Malnutrition in opium addiction due to economic problems and loss of appetite due to effect of opium can prone them to cardiovascular disease.^17^

In the present study, the odds of severe CAD were increased among current opium addicted patients but it was not statistically significant. There are only a few data available in this regard. In their cross sectional study in 2007, Sadeghian et al found that opium consumption is a significant risk factor for coronary artery disease after adjusting conventional cardiovascular risk factors. Moreover, the amount of opium consumption was significantly associated with the severity of coronary atherosclerosis, as measured by clinical vessel score in this study.^7^ Marmor et al in 2004 reported that longterm exposure to opiates may mitigate CAD severity and its fatal consequences. But this study was done in autopsies samples.^8^ Another study in Yazd showed that the prevalence of opium addiction among myocardial infarction patients was higher than general population.^9^ Azimzadeh and co-workers in 2005 did not find any relationship between history of opium addiction and myocardial infarction.^21^

### 

#### Limitations

The definition of addiction in the present study was based on history of the patients and their pretensions were not confirmed by paraclinic tests. This problem can lead to underestimation of the addiction, which can affect present results. The other shortcoming of this study is that coronary angiography is an invasive operation. Therefore, healthy subjects could not be entered into the study without any cardiac complaint as healthy controls due to ethical considerations.

## Conclusions

The findings indicated that current opium users - compared with non users - had a higher risk for severe CAD. But it was roughly confounded by other cofactors such as cigar smoking, age and sex. A doseresponse relationship was noticed between the type of opium consumption and the severity of CAD, but it was not significant. Also a significant relationship between age, diabetes, hyperlipidemia, gender and severity of coronary artery stenosis was found. There are only a few data in this regards, which suggests an urgent need to perform more precise studies.
